# Evaluation of the Appropriateness of Piperacillin-Tazobactam Prescription in Community-Acquired Pneumonia: A Tertiary-Center Experience

**DOI:** 10.7759/cureus.51385

**Published:** 2023-12-31

**Authors:** Ali Almajid, Ali Bazroon, Hassan Albarbari, Hashim M Al-Awami, Alzahraa AlAhmed, Omar M Bakhurji, Ghadah Alharbi, Fatemah Aldawood, Zainab AlKhamis, Mohammed Alqarni, Mohammed Alabdullah, Raghad Almutairi

**Affiliations:** 1 Department of Internal Medicine, King Fahad Specialist Hospital, Dammam, SAU; 2 Faculty of Medicine, King Abdulaziz University Hospital, Jeddah, SAU; 3 College of Medicine, Imam Abdulrahman Bin Faisal University, Dammam, SAU; 4 College of Medicine, Qassim University, Buraydah, SAU

**Keywords:** community-acquired pneumonia, broad-spectrum, antimicrobial resistance, inappropriate use, antimicrobial stewardship, piperacillin-tazobactam, tazocin

## Abstract

Background

Antimicrobial resistance (AMR) has been designated a public health crisis by the World Health Organization. AMR can lead to escalated healthcare costs, higher mortality rates, increased morbidity, and more frequent hospitalizations. This study aimed to retrospectively evaluate the appropriateness of Tazocin prescription for community-acquired pneumonia (CAP).

Methodology

We conducted a retrospective analysis of patients aged ≥18 years who were admitted with a diagnosis of CAP and administered intravenous Tazocin between November 2021 and October 2022. The primary objective was to assess the appropriateness of Tazocin prescriptions in patients with CAP.

Results

A total of 39 patients with CAP were included, with a mean age of 61 ± 17.36 years. Overall, 24 (61%) patients were male. The rate of inappropriate prescriptions of Tazocin was 66.6%. The incidence of inappropriate Tazocin prescription varied significantly among different medical specialties, with the highest rate observed in the oncology-palliative specialty (90%; p = 0.033).

Conclusions

Our study affirms the inclination of physicians to prescribe Tazocin for CAP without justifiable indications and highlights the unwarranted use of Tazocin for CAP across various medical specialties. This is evidenced by the notably high rate of inappropriate empirical prescriptions.

## Introduction

Tazocin, a combination of β-lactam/β-lactamase inhibitors, is a broad-spectrum antibiotic that is effective against a spectrum encompassing gram-positive, gram-negative, and anaerobic bacteria. Notably, Tazocin is distinguished for its antipseudomonal activity, positioning it as an integral component of empirical therapy for community-acquired pneumonia (CAP), particularly in cases presenting robust risk factors for pseudomonal infection, such as prior pseudomonal infection or colonization. The emergence of antimicrobial resistance (AMR) is considered a serious public health crisis [1], leading to increased health expenditure, morbidity, and mortality [2]. Despite the evolution of AMR through natural processes, the overprescription and misuse of antibiotics have drastically accelerated the global emergence of AMR [3].

A previous study evaluated Tazocin usage across four distinct hospitals in the United States, revealing that approximately 30% of prescriptions were deemed inappropriate. This inappropriateness is predominantly attributed to incorrect empirical indications, with a substantial proportion of empirical use pertaining to pneumonia in the general context [4]. Notably, a retrospective study conducted at a tertiary hospital in Qatar indicated that Tazocin’s application in CAP was consistently inappropriate in 100% of cases [5].

Given the ubiquity of CAP, the consequential risk associated with the imprudent application of antimicrobials on a public health scale, and the lack of comprehensive data regarding Tazocin prescription in CAP in Saudi Arabia, this study aimed to retrospectively evaluate the appropriateness of Tazocin prescription for CAP at King Fahad Specialist Hospital, Dammam, Saudi Arabia.

## Materials and methods

Study design and setting

This retrospective, observational, cross-sectional study was conducted at the King Fahad Specialist Hospital, a tertiary hospital in Al-Dammam, Saudi Arabia. This study was approved by the Ethics Committee of the Institutional Review Board (IRB) (registration number: IRB-IDD0011) on 26/01/2023 and the data were accessed on 28/01/2023. Due to the anonymous nature of the study, explicit consent was not deemed necessary for data acquisition.

Inclusion and exclusion criteria

All individuals aged ≥18 years who were admitted with a confirmed diagnosis of CAP and received intravenous Tazocin between November 2021 and October 2022 were included in the study. CAP was defined as new infiltrates on chest radiographs in conjunction with respiratory manifestations, including fever, dyspnea, sputum production, or cough. Patients lacking chest imaging, those with uncertain CAP diagnoses, and those with incomplete data were excluded from the analysis.

Study definitions

Severe CAP was defined according to the criteria established by the Infectious Diseases Society of America (IDSA) in 2007/2019. The appropriateness of a Tazocin prescription was determined based on the guidelines outlined by the IDSA and American Thoracic Society (ATS) in 2019 [6]. Tazocin prescription was considered appropriate if the patient received a pseudomonal dose and met any of the following criteria: isolation of *Pseudomonas aeruginosa*, previous isolation of *Pseudomonas aeruginosa*, severe CAP in conjunction with recent (within 90 days) hospitalization, or receiving parenteral antibiotics within 90 days, and the medication was subsequently adjusted in accordance with the culture results. Furthermore, a Tazocin prescription was deemed appropriate if a non-pseudomonal dose was administered when pseudomonal coverage was not indicated.

Data collection and analysis

Medical records were examined to extract patients’ demographic, baseline, and outcome data. Additionally, an assessment was performed to determine the appropriateness of utilizing Tazocin as an antibiotic therapy for CAP in accordance with the aforementioned criteria.

Statistical analysis was conducted using SPSS version 28 (IBM Corp., Armonk, NY, USA). Continuous data are presented as mean and standard deviation (SD) or median and interquartile range (IQR), while categorical data are presented as frequencies and percentages, as appropriate. Continuous data were compared using the independent sample Student’s t-test, whereas categorical data were compared using the chi-square or Fisher’s exact test, as appropriate to the underlying data.

## Results

The characteristics of the enrolled patients are summarized in Table 1. The results indicated a male predominance of 61% (24 out of 39), with participants exhibiting a mean age of 61 ± 17.36 years. A total of 38 (97%) patients received Tazocin and required hospitalization.

**Table 1:** Patient demographics. BMI: body mass index; HIV: human immunodeficiency virus

Characteristics		Frequency	Percent (%)
Age	26–38 years	3	7.7
	39–53 years	9	23.1
	53–74 years	15	38.5
	Above 75 years	12	30.8
	Mean	61.7	-
	Standard deviation	± 17.36	-
Gender	Male	24	61.5
	Female	15	38.5
Nationality	Saudi	38	97.4
	Non-Saudi	1	2.6
BMI	Underweight (<18.5)	6	15.4
	Normal (18.5–24.9)	14	35.9
	Overweight (≥25)	13	33.3
	Obese (≥30)	6	15.4
Breast cancer	Yes	3	7.7
	No	36	92.3
Colorectal cancer	Yes	3	7.7
	No	36	92.3
Lung metastasis	Yes	7	17.9
	No	32	82.1
Pulmonary conditions	Yes	7	17.9
	No	32	82.1
Congestive heart failure	Yes	2	5.1
	No	37	94.9
Hematopoietic stem cell transplant	Yes	2	5.1
	No	37	94.9
HIV	Yes	0	0.0
	No	39	100.0
Other malignancy	Liver malignancy	1	2.6
	Renal malignancy	2	5.1
	Leukemia	8	20.5
	Lymphoma	1	2.6
	Primary lung cancer	1	2.6
	Meningioma	1	2.6
	No other malignancy	24	61.5
Solid organ transplant	Renal transplant recipient	4	10.3
	Not a transplant patient	35	89.7

In the cohort, 46% (18 patients) had a documented history of recent parenteral antibiotic use within 90 days of admission (Table 2). In this subgroup, the majority, 10 (55.5%) patients, received the appropriate prescription of Tazocin.

**Table 2:** Risk factors for pseudomonal infection.

Risk factor	Total population (n = 39), n (%)	Appropriate, N (%)	Inappropriate, N (%)
Recent hospitalization within 90 days	16 (41)	8 (50)	8 (50)
Recent parenteral antibiotic within 90 days	17 (43.5)	9 (53)	8 (47)
Recent antibiotic use within 90 days	18 (46)	10 (55.5)	8 (44.4)
Prior isolation of *Pseudomonas aeruginosa*	4 (10)	3 (75)	1 (25)

Empirical therapy consisting of Tazocin was administered in 100% of the cases. When prescribed by emergency medicine physicians, the prescription was deemed inappropriate in 77% of the instances, as opposed to 60% when prescribed by the primary care team (Table 3).

**Table 3:** Emergency physician vs. primary team appropriateness of empiric piperacillin-tazobactam prescription. ED: Emergency Department

Variable	Category	Appropriate, N (%)	Inappropriate, N (%)	Total
Prescriber specialty	ED physician	2	7	9
		22.22%	77.78%	100.00%
	Primary Team	12	18	30
		40.00%	60.00%	100.00%

Overall, 26 (66.6%) patients received inappropriate Tazocin prescriptions. A statistically significant difference in the appropriateness of Tazocin prescription was observed among the various medical specialties (p = 0.033) (Table 4). Notably, the highest rate of appropriate prescriptions was noted among hemato-oncology patients, with an appropriate rate of 70%. In contrast, only 31% of internal medicine patients received an appropriate prescription of Tazocin.

**Table 4:** Appropriate vs. inappropriate piperacillin-tazobactam prescription among different specialities.

Primary team piperacillin-tazobactam prescriber	Appropriate, N (%)	Inappropriate, N (%)	P-value
Internal medicine	5 (31.25%)	11 (68.75%)	0.033
Oncology/palliative	1 (10.00%)	9 (90.00%)	
Hematology	7 (70.00%)	3 (30.00%)	
Other services	-	3 (100.00%)	

Furthermore, 61.5% of patients had non-severe CAP, and 75% of those patients received inappropriate piperacillin-tazobactam prescriptions (Table 5).

**Table 5:** Severity of CAP vs. appropriateness of piperacillin-tazobactam prescription. CAP: community-acquired pneumonia

Variable	Total population (n = 39), n (%)	Appropriate, N (%)	Inappropriate, N (%)
Severe	15 (38.4)	7 (46.6)	8 (53.3)
Non-severe	24 (61.5)	6 (25)	18 (75)

Most Instances where antibiotic prescriptions were inappropriate are related to the absence of *Pseudomonas *isolation in current or previous sputum culture (Table 6).

**Table 6:** Instances of inappropriate piperacillin-tazobactam prescription. CAP: community-acquired pneumonia

Reason of inappropriateness	Number of patients (%)
No previous or current isolation of *Pseudomonas*	17 (43.6)
No recent parenteral antibiotic use within 90 days	11 (28)
No recent hospitalization within 90 days	10 (25.6)
Antibiotics not changed according to culture result	17 (43.6)
Non-severe CAP	16 (41)

A statistically significant correlation was observed between the appropriateness of Tazocin prescription and the duration of intensive care unit (ICU) stay, with a p-value of 0.001, as presented in Table 7. Specifically, the mean duration of ICU stay was 5.7 ± 5.51 days for the group with inappropriate prescriptions, as opposed to 2.65 ± 3.56 days for those with appropriately prescribed Tazocin.

**Table 7:** Outcome data of appropriate vs. inappropriate piperacillin-tazobactam prescription. ICU: intensive care unit

Data		Appropriate	Inappropriate	P-value
Hospital length of stay	Mean	4.3	9.5	0.442
	Standard deviation	1.53	3.54	
ICU length of stay	Mean	2.65	5.7	0.001
	Standard deviation	3.56	5.51	
Required intubation	Yes, n/N (%)	4	6	0.875
		40.00%	60.00%	
Mortality	Yes, n/N (%)	5	5	0.487
		50.00%	50.00%	

## Discussion

As CAP is a frequently encountered disease in the emergency department, and Tazocin resistance is increasing [7], this study sought to investigate the appropriateness of prescribing Tazocin in CAP, which is the first study from the Kingdom of Saudi Arabia.

This study yielded three important results. First, there was a notable rate of inappropriate prescription of Tazocin, accounting for 66.6% of cases. Second, instances of inappropriate Tazocin prescription were significantly correlated with prolonged ICU stay (p = 0.001). Finally, a discernible variation was observed across various medical specialties concerning the appropriateness of Tazocin prescription in CAP, demonstrating statistical significance (p = 0.033).

The elevated incidence of inappropriately prescribed Tazocin has been substantiated in previous studies. Khan et al. observed a 100% incidence of inappropriate usage in a cohort of 43 patients with CAP [5]. In contrast, Al Saleh et al. found that only approximately 52% of antibiotic prescriptions for patients with pneumonia were considered inappropriate in a study designed to assess the appropriateness of prescribing carbapenems and Tazocin at a tertiary hospital [8].

The oncology-palliative patient group exhibited the highest incidence of inappropriate prescriptions at 90% (p = 0.033). This observation may be attributed to a potential lack of knowledge among physicians regarding specific indications for Tazocin prescription in cases of CAP. Alternatively, it may stem from a presumption that a broad-spectrum antibiotic would be the most effective approach for immunocompromised patients. In contrast, the hemato-oncology patient group displayed the highest rate of appropriate Tazocin prescriptions at 70%. This finding can be rationalized by the customary practice of involving the infectious disease team in guiding antimicrobial therapy, given the intricate nature of cases encountered in the hemato-oncology department. Moreover, hemato-oncology patients manifest febrile neutropenia in approximately 80% of cases, in contrast to a 10-40% incidence of solid organ malignancies [9,10]. The presence of febrile neutropenia is linked to an increased risk of severe CAP (OR = 10.24, CI = 3.48-30) in hemato-oncology patients, thereby augmenting the likelihood of receiving an appropriate prescription for Tazocin [11]. Furthermore, the mere presence of febrile neutropenia is an indication of a Tazocin prescription. Consequently, a high rate of appropriate prescriptions was expected among hemato-oncology patients given the higher likelihood of having risk factors for *Pseudomonas *infection and severe CAP in these patients.

Inappropriate prescriptions were more prevalent among emergency physicians (77%) than the primary care teams (60%). This aligns with a comparable observation made in an academic medical center’s emergency department, where 81% of 100 discharged patients receiving fluoroquinolone therapy received an inappropriate prescription. This inappropriateness stemmed from either fluoroquinolone not being the recommended initial antibiotic, or in cases where no infection was present [12].

Inappropriate prescriptions can cause significant short and long-term adverse effects. Notably, our study identified a notable increase in the mean length of ICU stay among the patients who received such prescriptions. This phenomenon may be attributed, in part, to alterations in the gut microbiota, which could arise as a secondary consequence of critical illness, particularly severe infections, and the administration of broad-spectrum antibiotics, such as Tazocin. Such alterations may lead to infectious complications, ultimately resulting in unfavorable patient outcomes [13]. Ojima et al. postulated that changes in the Bacteroidetes/Firmicutes ratio in ICU patients may serve as a prognostic indicator of patient outcomes. Their study, which focused on 12 ICU patients, of whom six succumbed, revealed significant shifts in the Bacteroidetes/Firmicutes ratio, exceeding either >10 or <0.01. Conversely, patients who survived did not exhibit drastic changes in this ratio [14]. Additionally, the prolonged ICU stay observed in our study may also be attributed to an elevated risk of *Clostridium difficile* and fungal infections in patients receiving inappropriate broad-spectrum antibiotics in ICU settings [15,16].

Strength and limitations

To our knowledge, this is the first report from Saudi Arabia to focus on the appropriateness of Tazocin prescription in CAP. Second, the collected variables were rigorously extracted using predetermined inclusion and exclusion criteria to minimize extraction errors and missing values. However, the retrospective nature of the study, which was a single-center study with a small sample size resulted in a limited generalizability to other healthcare facilities.

## Conclusions

Our study highlighted the inclination of physicians to prescribe Tazocin for CAP in the absence of justifiable indications, thereby revealing a notable incidence of inappropriate Tazocin prescriptions for CAP. The implications of these findings extend to the field of antimicrobial stewardship, advocating for the implementation of local clinical practice guidelines and underscoring the importance of adhering to the latest evidence. Such measures are crucial for mitigating the risk of the emergence of AMR, on both local and global scales.
